# Laparoscopic systemic devascularization of uterine cornu for cornual resection in interstitial pregnancy

**DOI:** 10.4274/tjod.23500

**Published:** 2015-09-15

**Authors:** Yakup Yalçın, Burak Tatar, Ebru Erdemoğlu, Mehmet Özgür Akkurt, And Yavuz, Evrim Erdemoğlu

**Affiliations:** 1 Süleyman Demirel University Faculty of Medicine, Department of Gynecologic Oncology, Isparta, Turkey; 2 Şifa Hospital, Clinic of Obstetrics and Gynecology, Isparta, Turkey; 3 Süleyman Demirel University Faculty of Medicine, Department of Obstetrics and Gynecology, Isparta, Turkey

**Keywords:** Cornual pregnancy, laparascopy, uterine artery occlusion, interstitial pregnancy

## Abstract

Cornual pregnancies carry a greater maternal mortality risk than ampullary ectopic pregnancies and they may cause significant hemorrhage. A woman aged 36 years with a six-week history of amenorrhea, slight vaginal bleeding, and low abdominal pain of three days duration presented to our clinic. A diagnosis of right cornual ectopic pregnancy was made using ultrasonographic findings. Laparoscopic exploration confirmed the diagnosis. We occluded the uterine artery at its origin and also transected vessels within the mesosalpinx and uteroovarian ligament to successfully accomplish avascularization of a cornual pregnancy. Occlusion of the uterine arteries is reported to be a safe and blood-sparing technique. Severe hemorragia may occur during the operation; therefore, techniques to minimize blood loss are reported. In our case, occlusion of the uterine artery and transection of the mesosalpinx and uteroovarian vessels provided a bloodless operation and there was no need to bilaterally occlude vessels.

## INTRODUCTION

Interstitial or cornual pregnancy occurs when an embryo is implanted in the proximal portion of the fallopian tube, which is within the muscular wall of the uterus. Cornual pregnancy is a rare event, accounting for 2-6% of all ectopic pregnancies^(1)^. It poses a significant diagnostic and therapeutic challenge and carries a greater maternal mortality risk than ampullary ectopic pregnancy. Cornual pregnancy tends to present relatively late, at 7-12 weeks of gestation, due to the intrinsic properties of the myometrium. Significant maternal hemorrhage, which leads to hypovolemia and shock, can rapidly result from cornual rupture. The mortality rate is 2-2.5%^(1)^. Improvements in ultrasound technology and increased experience with these unusual cases have allowed for the visualization of ectopic pregnancies with uncommon implantation sites at earlier stages.

The main treatments for ectopic pregnancy management are expectant management, medical treatment and surgical therapy, including laparotomy and laparoscopy. Unique to interstitial pregnancy is the location of the gestational sac in a highly vascular area, near the junction of the uterine and ovarian vessels. Consequently, there is a high risk of bleeding with resultant high morbidity and mortality. Therefore, to reduce the blood supply before cornual resection, we performed prophylactic selective ipsilateral uterine artery ligation at its origin, and uteroovarian vessel ligation using a laparoscopic approach.

## CASE REPORT

A G5 P3 A1 woman aged 36 years with a six-week history of amenorrhea, slight vaginal bleeding, and low abdominal pain of three days duration presented to our clinic. There was no significant disease or operations in her medical history. Her vital signs were stable and general physical examination was unremarkable. Pelvic examination revealed a closed cervix with a minimal blood clot in the vaginal vault. The uterus was of normal size and there was tenderness on palpation. The abdomen was not tender and there was no sign of acute abdomen.

A diagnosis of right cornual ectopic pregnancy was made using ultrasonographic findings. The ultrasound examination revealed an anteverted uterus, with an endometrial thickness of 4 mm. A gestational sac of 22x36 mm without yolk sac and fetal pole was observed in the right uterine cornual area. There was no free fluid in the cul-de-sac. Her hemoglobin level was 11.3 g/dL, and the β-HCG level was 16.928 mU/mL. The patient preferred her ectopic pregnancy to be managed with laparoscopic treatment rather than medically, and desired concurrent sterilization.

**Figure 1:**
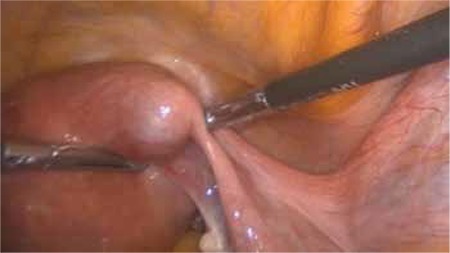
Right uterine cornual pregnancy

**Figure 2:**
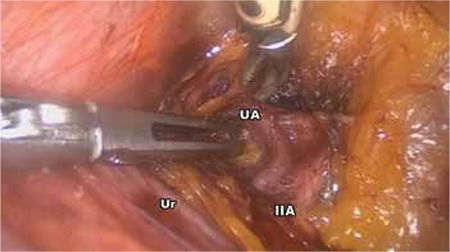
Occlusion of uterine artery at its origin UA: Uterine artery, IIA: Internal iliac artery, Ur: Ureter

**Figure 3:**
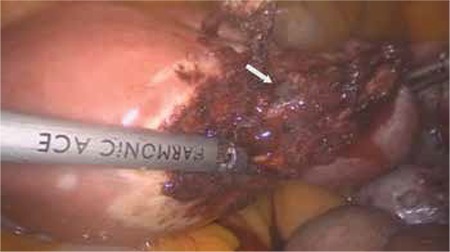
Cornuotomy was performed with minimal blood loss after occlusion of ipsilateral uterine artery, and transection of mesosalpinx and uteroovarian ligament, Arrow: Cornual pregnancy

**Figure 4:**
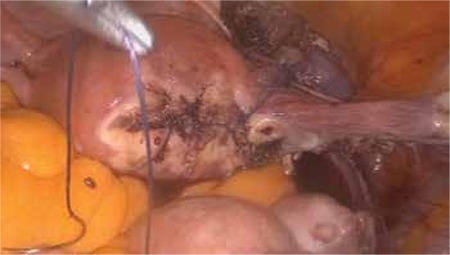
Metroplasty is performed with 2/0 polyglactin sutures Video 1. https://www.youtube.com/watch?v=LWb9juK3n2w

The patient was prepared in the Trendelenburg position with legs straight and arms tucked alongside the body. A 10 mm trocar was inserted through the umbilicus for endoscope access. Three 5 mm re-usable trocars were placed into the suprapubic area, and left and right lower abdomen. Exploration confirmed a cornual pregnancy in the right uterine cornu (Figure 1). The pelvic peritoneum over the psoas muscle was opened parallel to the right infundibulopelvic ligament, and then the external iliac artery, internal iliac artery, and ureter were identified. The right pararectal space was developed using blunt dissection between the right ureter and internal iliac artery. The uterine artery over the ureter was identified and isolated for a distance of 1.5-2 cm (Figure 2). The uterine artery was then coagulated by use of a bipolar grasper. The mesosalpinx of the right fallopian tube was coagulated and transected. A window was created above the ureter and then the uteroovarian ligament was coagulated and transected. The right cornu was avascularized and a resection of the uterine cornua was performed using a bipolar grasper and harmonic scalpel. The ectopic pregnancy was excised totally (Figure 3, Video 1). The incision site of the myometrium was sutured with intracorporeal 2/0 polyglactin sutures (Figure 4). Left tubal sterilization was completed using bipolar coagulation. The amount of bleeding was 10 mL during the operation.

The preoperative hemoglobin level of the patient was 11.3 g/dL and at the sixth postoperative hour the hemoglobin level was 11.2 g/dL. The patient recovered uneventfully and was discharged on the second postoperative day. Her HCG level was 2 mU/mL on day 15.

## DISCUSSION

Cornual pregnancy is a rare form of ectopic pregnancy that causes significant maternal morbidity and mortality. Cornual pregnancy remains one of the most difficult gestations to diagnose and treat. Cornual pregnancy is located in a highly vascular area, near the junction of the uterine and ovarian vessels. Rupture and/or surgical intervention in this area may result in catastrophic hemorrhage because of its rich blood supply. This is the first report in the literature to present ipsilateral occlusion of the uterine artery on the internal iliac and occlusion of uteroovarian vessels for complete avascularization of cornual pregnancy.

Treatment modalities of cornual pregnancy are either conservative medical treatment or surgical. Methotrexate has been used successfully in tubal ectopic pregnancies. The use of methotrexate has also been studied in cornual pregnancies, either as systemic treatment or local injection. The main disadvantage of medical treatment in cornual pregnancies is severe bleeding, which may lead to emergency hysterectomy or/and loss of the patient, in contrast to tubal ectopic pregnancies. In addition, methotrexate treatment of cornual pregnancy may require prolonged hospitalization and multi-dose treatment. The time for complete resolution ranges from 19 to 129 days^(2,3)^. Some 15-20% of patients treated with methotrexate may eventually need surgery^(4)^.

Traditionally, the treatment of cornual pregnancy has been cornual resection by laparotomy or hysterectomy^(5)^. Increased laparoscopic experience along with new electrosurgical units and technologies have led to improved laparoscopic treatment of cornual pregnancy. Severe hemorragia may occur during the operation; therefore, techniques to minimize blood loss are reported. The most common method is to inject vasopressin into the myometrium^(4,5)^. However, vasopressin is not available world-wide; it is not available and approved in Turkey, for example. Moreover, it provides a bloodless surgical field for only a short time due to its relatively short half-life. It may also cause angina, myocardial infarction, hypertension and bradycardia^(4)^. Occlusion of the ascending branch of the uterine artery may be performed to decrease bleeding,^(6)^ although it might not be completely successful^(4)^. Other methods reported to control bleeding are suture loop tourniquets, stapler or endoloop^(7)^. There is an intensive network of vascular supply to the uterus. We occluded the uterine artery at its origin and also transected vessels within the mesosalpinx and uteroovarian ligament to successfully accomplish avascularization of a cornual pregnancy. Occlusion of the uterine arteries takes about 8 minutes and is reported to be a safe and blood-sparing technique in laparoscopic hysterectomy and laparoscopic myomectomy^(8)^. No major complications are reported in using this technique, particularly in relation to the ureter. In our case, there was only 10 mL of blood loss during the operation and no transfusion was required.
